# Emotional intelligence and life satisfaction among health sciences students: a cross-sectional study

**DOI:** 10.3389/fpsyg.2026.1867996

**Published:** 2026-09-29

**Authors:** Grace Andrea Huaca Guevara, Adriana Edit Miniet Castillo, Patricio-Gabriel Fuentes

**Affiliations:** Facultad de Ciencias de la Salud, Universidad Técnica del Norte, Ibarra, Ecuador

**Keywords:** cross-sectional studies, Ecuador, emotional intelligence, personal satisfaction, students, health occupations

## Abstract

**Background:**

Perceived emotional intelligence has been associated with psychological wellbeing and may be particularly relevant among health sciences students exposed to academic and interpersonal demands. This study examined the association between perceived emotional intelligence and life satisfaction among health sciences students.

**Methods:**

A cross-sectional analytical study was conducted in January 2023 among 334 undergraduate students enrolled in Medicine, Nursing, Nutrition, and Physiotherapy at an Ecuadorian university. Perceived emotional intelligence was assessed using the 24-item Trait Meta-Mood Scale (TMMS-24), and life satisfaction was assessed using the five-item Satisfaction With Life Scale (SWLS). Age, sex, and academic program were included as covariates in the adjusted model. Group differences were examined using non-parametric tests, the association between the study variables using Spearman's rank correlation, and adjusted associations using multiple linear regression.

**Results:**

Perceived emotional intelligence was positively associated with life satisfaction (ρ = 0.383, *p* < 0.001). In the adjusted model, perceived emotional intelligence remained positively associated with life satisfaction (*B* = 0.194, 95% CI 0.125–0.263, β = 0.292, *p* < 0.001). Female students reported lower life satisfaction than male students (*B* = −2.145, 95% CI −3.982 to −0.308, *p* = 0.022), whereas no significant sex difference was observed in perceived emotional intelligence. No statistically significant differences in either construct were detected across academic programs or ethnic groups. The adjusted model explained 10.7% of the variance in life satisfaction (*R*^2^ = 0.107).

**Conclusion:**

Perceived emotional intelligence was positively associated with life satisfaction among Ecuadorian health sciences students after adjustment for age, sex, and academic program. The modest variance explained by the model supports a multifactorial interpretation of life satisfaction. Given the cross-sectional design, these findings should be interpreted as associations rather than causal relationships.

## 1 Introduction

Emotional intelligence (EI) has become an increasingly relevant construct for understanding psychological adjustment, interpersonal functioning, and wellbeing in educational settings. Within the ability model, EI encompasses interrelated capacities involved in perceiving, using, understanding, and managing emotional information (Mayer et al., 2024). However, EI can be assessed through different conceptual and measurement approaches. Self-report instruments such as the Trait Meta-Mood Scale (TMMS) assess individuals' perceptions of their own emotional functioning rather than objectively measured emotional abilities. The TMMS conceptualizes perceived emotional intelligence through three related domains: Emotional Attention, referring to the extent to which individuals attend to their emotional states; Emotional Clarity, reflecting their perceived ability to understand those states; and Emotional Repair, referring to their perceived capacity to regulate negative emotional states and maintain or restore positive ones Salovey et al., (1995). The Spanish modified version of the TMMS has demonstrated adequate psychometric properties in university students Fernández-Berrocal et al., (2004).

Life satisfaction represents the cognitive component of subjective wellbeing and refers to an individual's global evaluation of their life according to personally relevant standards. The Satisfaction With Life Scale (SWLS) was developed to assess this global cognitive judgment rather than specific affective experiences (Diener et al., 1985). The Spanish version has shown a unidimensional structure and adequate reliability (Vázquez et al., 2013). More recently, the psychometric properties of the SWLS were examined in 4,524 students from three Ecuadorian universities. The scale showed a well-fitting unidimensional structure, measurement invariance across sex, and adequate internal consistency (ω = 0.84), providing contemporary evidence supporting its use among Ecuadorian university students (López-Guerra et al., 2025).

Life satisfaction during university education is particularly relevant because students face simultaneous academic, interpersonal, financial, and developmental demands. Consequently, their evaluations of life are unlikely to depend on a single psychological characteristic. Socioemotional resources such as emotional intelligence, resilience, optimism, and perceived social support have been associated with life satisfaction in university populations (García-Martínez et al., 2023; Hidalgo-Fuentes et al., 2021). García-Martínez et al. (2023), for example, showed that emotional intelligence and resilience were directly associated with life satisfaction in university students, while optimism contributed to the relationships within their structural model. These findings support a multifactorial understanding of life satisfaction in which emotional functioning operates alongside other individual and social resources.

Within this framework, the association between perceived emotional intelligence and life satisfaction can be understood through complementary theoretical perspectives. The Ability Model of Emotional Intelligence conceptualizes emotional functioning in terms of capacities to perceive, use, understand, and manage emotional information (Mayer et al., 2024). Although the TMMS-24 does not directly assess these abilities, but rather individuals' perceptions of their emotional attention, clarity, and repair (Salovey et al., 1995; Fernández-Berrocal et al., 2004), greater perceived understanding and regulation of emotional experiences may be associated with more adaptive appraisal and management of emotionally demanding situations. These processes provide a conceptual basis for expecting perceived emotional functioning to be positively related to evaluations of life satisfaction.

This relationship can also be interpreted in light of Broaden-and-Build Theory (Fredrickson, 2001), which proposes that positive emotional experiences can broaden individuals' momentary thought-action repertoires and contribute over time to the development of enduring psychological and social resources. From this perspective, greater perceived emotional clarity and repair may facilitate recovery from negative emotional states and the maintenance of more adaptive emotional experiences, potentially supporting resources relevant to wellbeing and more favorable evaluations of life. This framework does not imply that emotional intelligence alone determines life satisfaction; rather, it is consistent with a multifactorial model in which emotional functioning operates alongside resilience, optimism, social support, coping resources, and contextual conditions.

Evidence accumulated across studies supports a positive association between emotional intelligence and subjective wellbeing. Previous research has consistently reported positive associations between perceived emotional intelligence and life satisfaction in university populations (Extremera and Fernández-Berrocal, 2005). A meta-analytic investigation by Sánchez-Álvarez et al. (2016) found an overall positive relationship between EI and subjective wellbeing across studies. Similarly, Cejudo et al. (2016), using the TMMS-24 and SWLS in 432 university students, found that life satisfaction was positively related to Emotional Clarity and Emotional Repair and also to resilience. More recent cross-cultural evidence involving 1,220 university students from Peru and Spain likewise showed significant associations between TMMS-24 dimensions and life satisfaction in both samples (Vilca-Pareja et al., 2023). Together, these findings suggest that perceived emotional functioning is consistently related to life satisfaction across different university populations, although the magnitude and specific dimensional patterns may vary across contexts.

Evidence from Latin America is particularly relevant because emotional experiences and evaluations of wellbeing occur within specific social, educational, and cultural environments. In Ecuador, Hidalgo-Fuentes et al. (2021) studied 381 university students and found that emotional intelligence and perceived social support were both related to life satisfaction, highlighting the simultaneous contribution of emotional and interpersonal resources. The cross-cultural findings reported by Vilca-Pareja et al. (2023) in Peruvian and Spanish students further suggest that the association between emotional intelligence and life satisfaction can be observed across different cultural contexts. These findings provide an important regional foundation while also indicating the need for further studies examining specific educational populations in Latin America.

The relationship between emotional functioning and wellbeing may be particularly relevant in health sciences education. Students preparing for health professions are exposed to substantial academic and interpersonal demands while developing competencies for communication, teamwork, clinical decision-making, empathy, and interaction with individuals experiencing illness and distress. A systematic review of EI among medical students found that higher EI was associated with several favorable educational and psychological characteristics, including better mental health, lower perceived stress and distress, greater empathy, and better academic performance (Edussuriya et al., 2022). Recent research also indicates that EI is relevant to motivation, stress management, teamwork, empathy, and professional development in medical education (El Tarhouny et al., 2026). These findings provide a specific rationale for investigating emotional functioning in health professions education rather than assuming that findings from general university populations necessarily characterize students preparing for clinical and patient-centered roles.

Recent evidence has also strengthened the direct connection between emotional intelligence and life satisfaction in populations relevant to health and university education. Among nursing students, Lugo Ramirez et al. (2026) reported significant positive associations between life satisfaction and the three TMMS-24 dimensions of Emotional Attention, Emotional Clarity, and Emotional Repair. Likewise, Gómez-López et al. (2026) found that perceived stress was negatively associated with life satisfaction among university students, whereas the dimensions of emotional intelligence were positively associated with it. Importantly, EI did not moderate the association between stress and life satisfaction in that study, reinforcing the view that life satisfaction is a multifactorial outcome and that emotional intelligence represents one component within a broader network of psychological and contextual influences.

Despite this growing body of evidence, several questions remain relevant. The positive association between emotional intelligence and life satisfaction is already well established, and the purpose of further research is therefore not simply to demonstrate its existence in additional samples. Rather, it is important to determine the magnitude and robustness of this association across sociocultural and educational contexts and to examine whether it persists when relevant demographic and academic characteristics are considered. Much of the available evidence has been generated outside Ecuador, although Latin American and Ecuadorian research is emerging (Hidalgo-Fuentes et al., 2021; Vilca-Pareja et al., 2023). Moreover, previous studies have frequently involved general university populations or students from individual disciplines, providing comparatively limited evidence across multiple health sciences programs within the same educational setting.

The Ecuadorian health sciences context provides a relevant setting in which to address these questions. Previous Ecuadorian research has demonstrated associations between emotional intelligence, social support, and life satisfaction in university students (Hidalgo-Fuentes et al., 2021), and recent evidence supports the psychometric suitability of the SWLS in Ecuadorian higher education (López-Guerra et al., 2025). However, evidence specifically characterizing the magnitude of the association between perceived emotional intelligence and life satisfaction across Medicine, Nursing, Nutrition, and Physiotherapy remains limited. Accordingly, the present study does not assume that perceived emotional intelligence is linked to life satisfaction through a single direct psychological pathway. Rather, the direct association examined here represents one component of a potentially more complex network involving emotional regulation, stress appraisal, coping, resilience, social support, and other individual and contextual resources. Because these potential mediating and moderating mechanisms were not measured in the present study, they could not be formally tested and should be addressed in future longitudinal and multivariable research.

Therefore, the present study aimed to examine the association between perceived emotional intelligence and life satisfaction among undergraduate students enrolled in Medicine, Nursing, Nutrition, and Physiotherapy at an Ecuadorian university. Specifically, the study sought to: (1) describe perceived emotional intelligence and life satisfaction in the study population; (2) examine the association between perceived emotional intelligence and life satisfaction; (3) assess differences in these variables according to sex, academic program, and ethnicity; and (4) determine whether perceived emotional intelligence remained associated with life satisfaction after adjustment for age, sex, and academic program. Based on the theoretical framework and previous empirical evidence (Extremera and Fernández-Berrocal, 2005; Sánchez-Álvarez et al., 2016; Cejudo et al., 2016; Vilca-Pareja et al., 2023; Lugo Ramirez et al., 2026; Gómez-López et al., 2026), two hypotheses were formulated: (H1) higher perceived emotional intelligence scores would be positively associated with higher life satisfaction; and (H2) the positive association between perceived emotional intelligence and life satisfaction would remain evident after adjustment for age, sex, and academic program.

## 2 Materials and methods

### 2.1 Study design and participants

Participants were recruited from the undergraduate programs in Medicine, Nursing, Nutrition, and Physiotherapy at the Faculty of Health Sciences. The research team visited classrooms during the data-collection period to inform students about the study and invite voluntary participation. A total of 345 students were invited to participate. Of these, 334 agreed to participate and were included in the study, corresponding to a participation rate of 96.8%; 11 students declined participation. Students who agreed to participate completed the questionnaire electronically using the institutional Microsoft Forms 365 platform. Demographic information collected through the questionnaire included age, sex, academic program, and self-identified ethnicity. Participation was voluntary. Data were collected in January 2023.

No a priori sample-size calculation was performed because recruitment was conducted within the framework of the institutional research project during the predefined data-collection period. To characterize the statistical sensitivity of the achieved sample, a retrospective sensitivity analysis was performed using G^*^Power 3.1. With *n* = 334, six predictors, α = 0.05, and 80% power, the minimum detectable overall effect for the primary multiple regression model was approximately Cohen's *f* ^2^ = 0.042. This analysis was conducted retrospectively to characterize the sensitivity of the achieved sample and should not be interpreted as an a priori power calculation.

### 2.2 Study variables and instruments

#### 2.2.1 Perceived emotional intelligence

Perceived emotional intelligence was assessed using the 24-item Spanish version of the Trait Meta-Mood Scale (TMMS-24), derived from the original Trait Meta-Mood Scale developed by (Salovey et al. 1995) and subsequently adapted and validated in Spanish by Fernández-Berrocal et al. (2004). The TMMS-24 is a self-report instrument designed to assess individuals' perceptions of their emotional functioning rather than objective emotional ability.

The instrument comprises 24 items distributed across three dimensions: Emotional Attention (items 1–8), which reflects the degree to which individuals attend to and value their emotional states; Emotional Clarity (items 9–16), which refers to perceived understanding of one's emotional experiences; and Emotional Repair (items 17–24), which reflects perceived ability to regulate negative emotional states and maintain or restore positive emotional states. Each item is rated on a 5-point Likert scale ranging from 1 to 5, with higher scores indicating greater levels of the corresponding perceived emotional dimension.

In the original scoring framework, each dimension is scored separately by summing its eight items, yielding subscale scores ranging from 8 to 40. The Spanish validation study reported adequate psychometric properties for the three-factor structure (Fernández-Berrocal et al., 2004), and more recent evidence has supported the factorial structure and internal consistency of the TMMS-24 in Latin American university populations, including Peruvian university students (Fernández-Berrocal et al., 2025).

In the present study, the prespecified primary analysis used the sum of all 24 items as an overall indicator of perceived emotional functioning. Higher total scores reflected higher overall levels of perceived emotional functioning. This analytical approach should not be interpreted as evidence that the TMMS-24 is unidimensional or that a global score is psychometrically preferable to its established three-domain structure.

The validated Spanish-language version was administered directly; therefore, no translation or back-translation procedure was performed by the research team. Likewise, no additional cultural adaptation of the instrument was undertaken for the present study. Although the TMMS-24 has demonstrated psychometric support in Spanish-speaking and Latin American university populations (Fernández-Berrocal et al., 2004, 2025), its factorial validity and measurement equivalence were not independently established in the present Ecuadorian sample.

#### 2.2.2 Life satisfaction

Life satisfaction was assessed using the Spanish-language version of the Satisfaction With Life Scale (SWLS), originally developed by Diener et al. (1985). The SWLS consists of five items designed to assess an individual's global cognitive evaluation of life satisfaction. Each item is rated on a seven-point Likert scale ranging from 1 (*strongly disagree*) to 7 (*strongly agree*), yielding a total score ranging from 5 to 35, with higher scores indicating greater life satisfaction.

The Spanish-language version has demonstrated adequate validity and reliability (Vázquez et al., 2013). More recently, psychometric evidence from 4,524 Ecuadorian university students supported the unidimensional structure of the SWLS, measurement invariance across sex, and adequate internal consistency (ω = 0.84) (López-Guerra et al., 2025). The Spanish-language version was administered directly in the present study; therefore, no translation or back-translation procedure was performed by the research team.

### 2.3 Data collection procedure

Data were collected in January 2023. The research team visited classrooms across the Faculty of Health Sciences to recruit students from the participating undergraduate programs. Data collection was conducted electronically using a questionnaire administered through the institutional Microsoft Forms 365 platform. Participation was voluntary, and informed consent was obtained from all participants prior to completing the questionnaire. Confidentiality of the information collected was maintained throughout the study.

### 2.4 Statistical analysis

Data were analyzed using IBM SPSS Statistics version 27. Data were screened for completeness and consistency before analysis. Categorical variables were summarized as frequencies and percentages. Continuous variables were summarized using means and standard deviations, with medians and interquartile ranges additionally reported for non-parametric group comparisons.

The distributions of the main continuous variables were examined using the Kolmogorov-Smirnov test. The perceived emotional intelligence score did not show a statistically significant departure from normality (*p* = 0.097), whereas the life satisfaction score did (*p* < 0.001). Accordingly, non-parametric methods were used for the bivariate analyses involving these variables. Differences according to sex were examined using the Mann-Whitney *U* test, while differences according to academic program and ethnicity were assessed using the Kruskal-Wallis test. Effect sizes were reported alongside these comparisons: for Mann-Whitney *U* tests, effect size was calculated as *r* = |*Z*|/√*N*, whereas epsilon-squared (ε^2^) was used for Kruskal-Wallis tests. The association between perceived emotional intelligence and life satisfaction was evaluated using Spearman's rank correlation coefficient (ρ). A 95% bias-corrected and accelerated (BCa) bootstrap confidence interval for Spearman's ρ was estimated using 5,000 bootstrap samples.

To examine whether perceived emotional intelligence remained associated with life satisfaction after adjustment for selected covariates, a multiple linear regression model was fitted with life satisfaction as the dependent variable and perceived emotional intelligence as the main independent variable. Age, sex, and academic program were included as covariates. Age, sex, and academic program were included as covariates because they represented prespecified demographic and academic characteristics available in the original dataset that could plausibly be associated with students' emotional functioning and life satisfaction. Covariate selection was therefore conceptually informed rather than based on data-driven statistical screening.

Academic program was represented by dummy variables, with Medicine as the reference category.

Although the raw life satisfaction score showed a statistically significant departure from normality, multiple linear regression was retained because normality of the dependent variable itself is not a requirement of ordinary least squares regression. Model adequacy was therefore evaluated through diagnostic assessment of the residuals and other regression assumptions. Linearity and homoscedasticity were examined using plots of standardized residuals against standardized predicted values, and residual normality was assessed using a histogram and normal probability plot. Independence of residuals was assessed using the Durbin-Watson statistic, multicollinearity using tolerance and variance inflation factors (VIF), and potentially influential observations using Cook's distance and leverage values. Regression results were reported as unstandardized coefficients (*B*), standard errors (*SE*), standardized coefficients (β), 95% confidence intervals (CI), and *p*-values. Model fit was summarized using *R*^2^, adjusted *R*^2^, and the *F* statistic. All statistical tests were two-sided, with *p* < 0.05 considered statistically significant. Given the cross-sectional design, regression coefficients were interpreted as adjusted associations rather than causal effects.

At the Reviewer's request, exploratory interaction analyses were additionally conducted to examine whether the association between perceived emotional intelligence and life satisfaction varied by sex or academic program. Perceived emotional intelligence was mean-centered before constructing interaction terms. Sex was dummy-coded as male = 0 and female = 1, and academic program was represented by three dummy variables with Medicine as the reference category. Interaction terms were added hierarchically after the corresponding main effects and covariates. Changes in explained variance (Δ*R*^2^) and the regression coefficients and 95% confidence intervals of the interaction terms were examined. Because these analyses were not prespecified, they were considered exploratory and interpreted cautiously.

Because perceived emotional intelligence and life satisfaction were assessed simultaneously using self-report measures, an exploratory Harman's single-factor test was conducted to assess whether a single common component dominated the item-level variance. All 29 items from the TMMS-24 and SWLS were entered into an unrotated principal component analysis constrained to a single component. This analysis was considered a diagnostic assessment and not a definitive test for common method bias.

In response to peer review, supplementary exploratory analyses were conducted using the three TMMS-24 dimensions (Emotional Attention, Emotional Clarity, and Emotional Repair). Spearman's rank correlations were estimated between each dimension and life satisfaction, followed by a multiple linear regression model in which the three dimensions were entered simultaneously, adjusting for age, sex, and academic program. These analyses were not prespecified and were therefore considered exploratory and hypothesis-generating.

### 2.5 Reliability analysis

Internal consistency of the scores used in the present study was assessed using Cronbach's alpha. The 24 TMMS-24 items included in the overall perceived emotional intelligence score showed a Cronbach's α of 0.752 (95% CI [0.712, 0.789]), while the five-item SWLS showed a Cronbach's α of 0.845 (95% CI [0.817, 0.870]). These coefficients correspond to the scores used in the primary statistical analyses and were interpreted as indicators of internal consistency only, not as evidence of unidimensionality, construct validity, or cross-cultural measurement validity. The 95% confidence intervals for Cronbach's alpha were estimated using Feldt's method (Feldt et al., 1987).

For the supplementary exploratory dimension-specific analyses, internal consistency was also examined separately for the three established TMMS-24 dimensions. Cronbach's α was 0.665 for Emotional Attention, 0.284 for Emotional Clarity, and 0.518 for Emotional Repair. These estimates indicated heterogeneous and limited internal consistency across the dimension scores in the present sample, particularly for Emotional Clarity and Emotional Repair. Accordingly, dimension-specific analyses were interpreted cautiously and considered exploratory. No TMMS-24 items were removed or rescored *post hoc* to improve internal consistency.

### 2.6 Ethical considerations

The study was conducted in accordance with the ethical principles of the Declaration of Helsinki. Ethical approval was obtained from the Research Ethics Committee of Universidad Técnica del Norte, Ibarra, Ecuador, before data collection. Participation was voluntary, and informed consent was obtained from all participants prior to completion of the questionnaire. Confidentiality and anonymity were maintained throughout data collection, management, and analysis.

## 3 Results

Of the 345 students invited to participate, 334 agreed and were included in the analysis, corresponding to a participation rate of 96.8%. No missing data were present for the variables included in the primary analyses.

### 3.1 Sample characteristics

A total of 334 health sciences students were included in the analysis. The mean age of participants was 21.84 years (*SD* = 2.24). Most participants were female (*n* = 268, 80.2%), whereas 66 (19.8%) were male. Participants were enrolled in Nutrition (*n* = 92, 27.5%), Nursing (*n* = 90, 26.9%), Medicine (*n* = 88, 26.3%), and Physiotherapy (*n* = 64, 19.2%). Regarding self-identified ethnicity, 274 participants (82.0%) identified as Mestizo, 38 (11.4%) as Indigenous, and 22 (6.6%) as Afro-descendant (Table 1).

**Table 1:** Sociodemographic characteristics.

Variable	*n* (%)
Sex	
Male	66 (19.8%)
Female	268 (80.2%)
Age (years)	21.84 ± 2.24
Academic program	
Medicine	88 (26.3%)
Nursing	90 (26.9%)
Nutrition	92 (27.5%)
Physiotherapy	64 (19.2%)
Ethnicity	
Mestizo	274 (82.0%)
Indigenous	38 (11.4%)
Afro-descendant	22 (6.6%)

Percentages may not total 100% due to rounding.

### 3.2 Descriptive analysis

The mean overall TMMS-24 score used in the primary analyses was 84.32 (*SD* = 10.36), with observed scores ranging from 58 to 120. The mean life satisfaction score was 23.84 (*SD* = 6.87), with observed scores ranging from 5 to 35 (Table 2).

**Table 2:** Descriptive statistics and internal consistency of the study measures (*n* = 334).

Measure	Items	Mean	SD	Observed range	Cronbach's α (95% CI)
TMMS-24 overall score used in the primary analysis	24	84.32	10.36	58–120	0.752 [0.712,0.789]
Satisfaction With Life Scale (SWLS)	5	23.84	6.87	5–35	0.845 [0.817,0.870]

TMMS-24, Trait Meta-Mood Scale-24; SWLS, Satisfaction With Life Scale. The TMMS-24 comprises three dimensions: Emotional Attention, Emotional Clarity, and Emotional Repair. The overall 24-item score reported here corresponds to the score used in the primary analyses of the present study.

Internal consistency analyses conducted in the present sample yielded a Cronbach's α of 0.752 (95% CI [0.712, 0.789]) for the 24 TMMS-24 items included in the primary analysis and 0.845 (95% CI [0.817, 0.870]) for the five-item SWLS.

### 3.3 Association between perceived emotional intelligence and life satisfaction

A positive association was observed between perceived emotional intelligence and life satisfaction (Spearman's ρ = 0.383, 95% BCa bootstrap CI [0.276, 0.486], *p* < 0.001), indicating a moderate association (Table 3).

**Table 3:** Correlation between perceived emotional intelligence and life satisfaction (*n* = 334).

Variables	1	2
1. Perceived emotional intelligence	1.000	
2. Life satisfaction	0.383	1.000

Values are Spearman's rank correlation coefficients (ρ). For the association between perceived emotional intelligence and life satisfaction, 95% BCa bootstrap CI [0.276, 0.486], based on 5,000 bootstrap samples. *p* < 0.001, two-tailed.

### 3.4 Group comparisons

No statistically significant difference in perceived emotional intelligence was observed between male and female students (median [Q1–Q3]: 84.0 [78.0–90.5] vs. 84.5 [77.0–91.0], respectively; *U* = 8352, *p* = 0.484). In contrast, life satisfaction differed significantly by sex, with male students showing a higher median score than female students (27.0 [20.0–31.3] vs. 24.0 [18.0–29.0], respectively; *U* = 7188, *p* = 0.018).

No statistically significant differences across academic programs were observed for perceived emotional intelligence (*H* = 4.987, *p* = 0.173) or life satisfaction (*H* = 2.167, *p* = 0.539). Similarly, perceived emotional intelligence (*H* = 4.585, *p* = 0.101) and life satisfaction (*H* = 1.421, *p* = 0.491) did not differ significantly across ethnic groups (Table 4).

**Table 4:** Comparisons of perceived emotional intelligence and life satisfaction according to participant characteristics (*n* = 334).

Characteristic	*n*	Perceived emotional intelligence, median (Q1–Q3)	Test statistic/effect size	*p*	Life satisfaction, median (Q1–Q3)	Test statistic/effect size	*p*
Sex			*U* = 8352; *r* = 0.04	0.484		*U* = 7188; *r* = 0.13	0.018
Male	66	84.0 (78.0–90.5)			27.0 (20.0–31.25)		
Female	268	84.5 (77.0–91.0)			24.0 (18.0–29.0)		
Academic program			*H* = 4.987; ε^2^ = 0.006	0.173		*H* = 2.167; ε^2^ < 0.001	0.539
Medicine	88	85.5 (75.25–90.0)			23.0 (18.0–29.0)		
Nursing	90	83.0 (77.0–90.0)			25.0 (18.0–30.0)		
Nutrition	92	84.5 (78.0–91.0)			24.0 (20.0–29.0)		
Physiotherapy	64	86.0 (80.0–97.75)			26.0 (20.5–30.0)		
Ethnicity			*H* = 4.585; ε^2^ = 0.008	0.101		*H* = 1.421; ε^2^ < 0.001	0.491
Mestizo	274	84.0 (77.75–91.0)			25.0 (19.0–30.0)		
Indigenous	38	81.0 (72.0–88.0)			24.0 (20.0–30.0)		
Afro-descendant	22	85.0 (80.0–91.0)			22.0 (17.0–27.0)		

Values are presented as median (Q1–Q3), where Q1 and Q3 represent the 25th and 75th percentiles, respectively. Sex comparisons were performed using the Mann-Whitney *U* test, with effect size calculated as *r* = |*Z*|/√*N*. Academic program and ethnicity comparisons were performed using the Kruskal-Wallis *H* test, with effect size estimated using epsilon-squared (ε^2^). All tests were two-tailed, with statistical significance set at *p* < 0.05.

### 3.5 Regression analysis

The multiple linear regression model was statistically significant, *F*(6, 327) = 6.516, *p* < 0.001, and explained 10.7% of the variance in life satisfaction (*R*^2^ = 0.107; adjusted *R*^2^ = 0.090).

Perceived emotional intelligence remained positively associated with life satisfaction after adjustment for age, sex, and academic program (*B* = 0.194, *SE* = 0.035, β = 0.292, 95% CI [0.125, 0.263], *p* < 0.001). Female sex was negatively associated with life satisfaction relative to male sex (*B* = −2.145, *SE* = 0.934, β = −0.125, 95% CI [−3.982, −0.308], *p* = 0.022). Age was not significantly associated with life satisfaction (*B* = 0.040, *SE* = 0.162, β = 0.013, 95% CI [−0.279, 0.359], *p* = 0.805).

Academic program was entered as a categorical variable using indicator variables, with Medicine as the reference category. Compared with Medicine, no statistically significant differences in life satisfaction were observed for Nursing (*B* = 0.242, *p* = 0.808), Nutrition (*B* = −0.059, *p* = 0.953), or Physiotherapy (*B* = 0.757, *p* = 0.488) (Table 5).

**Table 5:** Multiple linear regression analysis of factors associated with life satisfaction (*n* = 334).

Predictor	*B*	*SE*	β	95% CI for *B*	*p*
Perceived emotional intelligence	0.194	0.035	0.292	0.125 to 0.263	< 0.001
Female sex (ref: male)	−2.145	0.934	−0.125	−3.982 to −0.308	0.022
Age (years)	0.040	0.162	0.013	−0.279 to 0.359	0.805
Nursing (ref: medicine)	0.242	0.996	0.016	−1.717 to 2.201	0.808
Nutrition (ref: medicine)	−0.059	0.985	−0.004	−1.997 to 1.880	0.953
Physiotherapy (ref: medicine)	0.757	1.090	0.043	−1.386 to 2.901	0.488

Dependent variable: Satisfaction With Life Scale total score. *B*, unstandardized regression coefficient; *SE*, standard error; β, standardized regression coefficient; CI, confidence interval. Medicine and male sex were the reference categories. Model statistics: *R*^2^ = 0.107; adjusted *R*^2^ = 0.090; *F*(6,327) = 6.516, *p* < 0.001; Durbin-Watson = 1.894.

Model diagnostics did not indicate problematic multicollinearity (VIF range = 1.025–1.521) or highly influential observations (maximum Cook's distance = 0.112). The Durbin-Watson statistic was 1.894, and centered leverage values ranged from 0.009 to 0.066. Standardized residuals ranged from −3.819 to 2.094. Although a small number of observations warranted diagnostic examination based on residual or leverage values, Cook's distance did not indicate undue influence on the fitted model. Visual inspection of the standardized residual histogram, normal probability plot, and standardized residuals vs. predicted values did not indicate major departures from the assumptions of the linear regression model. No observations were excluded on the basis of these diagnostics.

### 3.6 Supplementary exploratory dimension-specific analyses

In supplementary exploratory analyses, all three TMMS-24 dimensions were positively associated with life satisfaction at the bivariate level. Emotional Attention showed the strongest association (Spearman's ρ = 0.373, *p* < 0.001), followed by Emotional Repair (ρ = 0.291, *p* < 0.001) and Emotional Clarity (ρ = 0.205, *p* < 0.001).

When the three TMMS-24 dimensions were entered simultaneously into a multiple linear regression model adjusted for age, sex, and academic program, the overall model was statistically significant, *F*(8, 325) = 6.779, *p* < 0.001, with *R*^2^ = 0.143 and adjusted *R*^2^ = 0.122. Emotional Attention was positively associated with life satisfaction (*B* = 0.482, *SE* = 0.092, β = 0.348, 95% CI [0.302, 0.662], *p* < 0.001). Emotional Clarity (*B* = −0.086, *SE* = 0.107, β = −0.047, 95% CI [−0.296, 0.124], *p* = 0.419) and Emotional Repair (*B* = 0.056, *SE* = 0.111, β = 0.033, 95% CI [−0.162, 0.273], *p* = 0.614) were not independently associated with life satisfaction in this mutually adjusted model. The variance inflation factors ranged from 1.025 to 1.660, and the Durbin-Watson statistic was 1.924.

These dimension-specific analyses were not prespecified and should therefore be regarded as exploratory and hypothesis-generating. Their interpretation is further limited by the heterogeneous internal consistency of the TMMS-24 dimension scores observed in the present sample. These results are summarized in Table 6.

**Table 6:** Supplementary exploratory analyses of TMMS-24 dimensions and life satisfaction (*n* = 334).

TMMS-24 dimension	Mean (SD)	Cronbach's α	Spearman's ρ with life satisfaction	*B*	*SE*	β	95% CI for *B*	*p*
Emotional attention	29.58 (4.96)	0.665	0.373	0.482	0.092	0.348	[0.302, 0.662]	< 0.001
Emotional clarity	27.07 (3.75)	0.284	0.205	−0.086	0.107	−0.047	[−0.296, 0.124]	0.419
Emotional repair	27.67 (4.09)	0.518	0.291	0.056	0.111	0.033	[−0.162, 0.273]	0.614

Spearman's correlations represent bivariate associations between each TMMS-24 dimension and life satisfaction; all three correlations were statistically significant at *p* < 0.001. Regression coefficients were obtained from a supplementary exploratory multiple linear regression model in which Emotional Attention, Emotional Clarity, and Emotional Repair were entered simultaneously and adjusted for age, sex, and academic program (Medicine as the reference category). Model statistics: *F*(8, 325) = 6.779, *p* < 0.001; *R*^2^ = 0.143; adjusted *R*^2^ = 0.122; Durbin-Watson = 1.924; VIF range = 1.025–1.660. Dimension-specific analyses were not prespecified and should be considered exploratory and hypothesis-generating. B, unstandardized regression coefficient; SE, standard error; β, standardized regression coefficient; CI, confidence interval.

### 3.7 Exploratory interaction analyses

Exploratory analyses suggested that the strength of the association between perceived emotional intelligence and life satisfaction differed by sex. Adding the EI × sex interaction increased explained variance from *R*^2^ = 0.107 to *R*^2^ = 0.140 (Δ*R*^2^ = 0.033). The interaction term was statistically significant (*B* = −0.383, 95% CI [−0.597, −0.169], *p* < 0.001), indicating a stronger positive EI-life satisfaction association among male than female students.

Exploratory analyses also suggested that the strength of the association may differ across academic programs. Adding the EI × academic program interaction terms increased explained variance from *R*^2^ = 0.107 to *R*^2^ = 0.149 (Δ*R*^2^ = 0.042). Relative to Medicine, the EI-life satisfaction slope was significantly weaker in Nursing (Binteraction = −0.323, 95% CI [−0.549, −0.097], *p* = 0.005) and Nutrition (Binteraction = −0.358, 95% CI [−0.546, −0.171], *p* < 0.001), whereas the difference for Physiotherapy did not reach statistical significance (Binteraction = −0.168, 95% CI [−0.356, 0.020], *p* = 0.079). Given their *post hoc* exploratory nature, the unequal subgroup sizes, and the absence of established TMMS-24 measurement invariance across these subgroups, these interaction findings should be considered hypothesis-generating only and should not be interpreted as established subgroup differences.

### 3.8 Exploratory assessment of common method variance

In the exploratory assessment of common method variance, the first unrotated component accounted for 19.9% of the total variance across the 29 TMMS-24 and SWLS items. This finding did not indicate dominance of a single component; however, Harman's single-factor procedure cannot rule out common method bias.

## 4 Discussion

The present study does not challenge the well-established positive association between emotional intelligence and life satisfaction; rather, it provides context-specific evidence regarding its magnitude and robustness among Ecuadorian health sciences students. The observed association was moderate (Spearman's ρ = 0.383) and remained statistically significant after adjustment for age, sex, and academic program. Importantly, however, the multivariable model explained only 10.7% of the variance in life satisfaction, indicating that perceived emotional intelligence represents one component of a broader and multifactorial set of correlates of students' life satisfaction.

The positive association observed is consistent with previous research in university populations. Extremera and Fernández-Berrocal (2005) reported that perceived emotional intelligence, particularly Emotional Clarity and Emotional Repair, was associated with greater life satisfaction. Similarly, the meta-analysis by Sánchez-Álvarez et al. (2016) identified a positive relationship between emotional intelligence and subjective wellbeing, including its cognitive component. Studies using the TMMS-24 and SWLS have also reported positive relationships between emotional intelligence dimensions and life satisfaction among university students (Cejudo et al., 2016; Vilca-Pareja et al., 2023). More recently, Lugo Ramirez et al. (2026) found positive associations between life satisfaction and Emotional Attention, Emotional Clarity, and Emotional Repair among nursing students. Although these studies differ in populations and scoring approaches, their findings are broadly consistent with the positive association identified in the present study.

Several psychological processes may contribute to this relationship. Greater perceived ability to understand and regulate emotional experiences may facilitate appraisal of demanding situations, adaptive coping, and recovery from negative emotional states, which may in turn be associated with more favorable evaluations of life. Previous TMMS-based studies have particularly highlighted Emotional Clarity and Emotional Repair in relation to life satisfaction (Extremera and Fernández-Berrocal, 2005; Cejudo et al., 2016). Emotional functioning, however, is unlikely to operate independently. García-Martínez et al. (2023) found that emotional intelligence and resilience were directly associated with life satisfaction and that optimism also contributed within their structural model. In Ecuadorian university students, Hidalgo-Fuentes et al. (2021) identified emotional intelligence and perceived social support as relevant to life satisfaction. Thus, the present association should be understood within a broader network of psychological and social resources. Because coping, resilience, optimism, and social support were not measured in the present study, these mechanisms remain plausible explanations rather than conclusions derived from our data. This interpretation is also compatible with Broaden-and-Build Theory (Fredrickson, 2001), according to which adaptive positive emotional experiences may contribute to the development and maintenance of psychological and social resources relevant to wellbeing.

The supplementary dimension-specific analyses provide additional, although exploratory, context for the overall association observed in this study. All three TMMS-24 dimensions were positively associated with life satisfaction at the bivariate level; however, when Emotional Attention, Emotional Clarity, and Emotional Repair were entered simultaneously with the study covariates, only Emotional Attention remained independently associated with life satisfaction. This finding should not be interpreted as establishing Emotional Attention as the principal or dominant TMMS-24 dimension associated with life satisfaction. The analyses were not prespecified, and the internal consistency of the dimension scores was limited in the present sample, particularly for Emotional Clarity and Emotional Repair. Accordingly, these dimension-specific findings should be considered hypothesis-generating and require replication with stronger psychometric support.

The findings are particularly relevant to health sciences education, where students face academic demands while progressively developing competencies involving communication, teamwork, empathy, decision-making, and interaction with individuals experiencing illness and distress. A systematic review among medical students identified associations between emotional intelligence and psychological and educational outcomes, including mental health, perceived stress, empathy, and academic performance (Edussuriya et al., 2022). Emotional intelligence has also been linked conceptually to professional development and identity formation in medical education (El Tarhouny et al., 2026). In Ecuador, previous research has already demonstrated an association between emotional intelligence and life satisfaction among university students (Hidalgo-Fuentes et al., 2021). Therefore, the contribution of the present study is not to establish this relationship for the first time in Ecuador, but to examine it in a multidisciplinary health sciences sample comprising Medicine, Nursing, Nutrition, and Physiotherapy students and to evaluate whether the association remained after adjustment for selected demographic and academic characteristics.

The Ecuadorian context should also be considered when interpreting these findings. Emotional expression, self-perceptions of emotional functioning, and evaluations of life satisfaction are embedded within social and cultural environments, and their meaning or expression may vary across populations. Although the present study provides evidence from an underrepresented Latin American educational context, it did not directly assess cultural values or culturally specific mechanisms. Accordingly, differences or similarities between the present findings and those reported in other countries cannot be attributed to culture on the basis of these data alone. Cross-cultural and multicenter studies using measurement-equivalent instruments are needed to determine the extent to which the magnitude and pattern of the EI-life satisfaction association generalize across sociocultural settings.

A notable finding was the difference in life satisfaction according to sex. Male students had a higher median life satisfaction score than female students (27.0 vs. 24.0), and female sex remained negatively associated with life satisfaction after adjustment (*B* = −2.145, 95% CI −3.982 to −0.308, *p* = 0.022). In contrast, perceived emotional intelligence did not differ significantly by sex. Previous university research has also reported higher life satisfaction among male students, although sex-related patterns in emotional intelligence and wellbeing have not been consistent across studies (Vilca-Pareja et al., 2023; Gavín-Chocano et al., 2020). Recent evidence further suggests that sex may be relevant to relationships between emotional functioning and psychological outcomes. Vo et al. (2024), for example, found no significant overall difference in emotional intelligence by sex but observed sex-related differences in its associations with depression, anxiety, and stress among undergraduates. Their outcomes and EI instrument differed from those used here, so these findings cannot directly explain the present sex difference. Furthermore, women represented 80.2% of our sample, and potentially relevant factors such as perceived stress, social support, socioeconomic circumstances, family responsibilities, and psychological symptoms were not assessed.

Exploratory interaction analysis further suggested that the strength of the association between perceived emotional intelligence and life satisfaction may differ by sex. The EI × sex interaction was statistically significant (*B* = −0.383, 95% CI −0.597 to −0.169, *p* < 0.001), with the positive association appearing stronger among male than female students. This finding should be considered hypothesis-generating rather than confirmatory because the interaction analysis was not prespecified and the sex distribution was markedly unequal. Furthermore, measurement equivalence of the TMMS-24 across sex was not established in the present sample. The sex-related findings should therefore be interpreted cautiously and require replication in more balanced samples.

No statistically significant differences in perceived emotional intelligence or life satisfaction were detected across academic programs or ethnic groups. These results indicate an absence of statistically detectable differences in the present sample rather than equivalence between groups. This distinction is especially important for ethnicity because the sample was predominantly Mestizo (82.0%), with substantially smaller Indigenous and Afro-descendant groups. Likewise, potential differences between academic programs related to year of study, workload, curricular characteristics, or clinical exposure were not examined. Studies designed and adequately powered for subgroup comparisons are needed before drawing stronger conclusions regarding program- or ethnicity-related differences. Notably, the absence of statistically significant overall differences across academic programs does not imply that the association between perceived emotional intelligence and life satisfaction was identical across programs. In exploratory interaction analyses, the EI-life satisfaction association was significantly weaker in Nursing (Binteraction = −0.323, 95% CI −0.549 to −0.097, *p* = 0.005) and Nutrition (Binteraction = −0.358, 95% CI −0.546 to −0.171, *p* < 0.001) relative to Medicine, whereas the difference for Physiotherapy did not reach statistical significance (Binteraction = −0.168, 95% CI −0.356 to 0.020, *p* = 0.079). Because these analyses were *post hoc*, subgroup sizes were unequal, and measurement invariance of the TMMS-24 across academic programs was not established in the present sample, these findings should be regarded as hypothesis-generating signals requiring independent replication rather than evidence of established program-specific differences.

Perceived emotional intelligence remained associated with life satisfaction in the adjusted model, while the complete model explained 10.7% of its variance (*R*^2^ = 0.107; adjusted *R*^2^ = 0.090). This modest explained variance reinforces the multifactorial nature of life satisfaction rather than diminishing the observed association. Previous research has identified additional contributors, including perceived stress (Gómez-López et al., 2026), resilience and optimism (García-Martínez et al., 2023), perceived social support (Hidalgo-Fuentes et al., 2021), and emotion-regulation and economic factors (Vilca-Pareja et al., 2026). Accordingly, perceived emotional intelligence should be interpreted as one relevant correlate within a broader network of individual, interpersonal, academic, and contextual factors rather than as a dominant determinant of life satisfaction. From an educational perspective, emotional functioning may therefore represent one component of broader student wellbeing initiatives, but the moderate magnitude of the association and limited explained variance do not support treating emotional intelligence as a stand-alone intervention target. Student wellbeing strategies should also consider factors such as stress, social support, resilience, academic demands, and socioeconomic circumstances. Moreover, because the present study was cross-sectional, it cannot establish that increasing emotional intelligence would improve life satisfaction; this possibility requires longitudinal and intervention research.

The direct association examined in the present study should therefore be interpreted as a parsimonious statistical representation of a potentially more complex set of pathways rather than as evidence of a single EI-to-life-satisfaction mechanism.

### 4.1 Strengths and limitations

This study included students from four health sciences programs and examined the association between perceived emotional intelligence and life satisfaction using both bivariate and adjusted analyses. The SWLS showed good internal consistency in the present sample (Cronbach's α = 0.845), consistent with recent psychometric evidence supporting its use among Ecuadorian university students (López-Guerra et al., 2025).

Several limitations should be considered. First, the cross-sectional design precludes establishing temporal ordering or causal direction. Although perceived emotional intelligence was associated with life satisfaction after adjustment for selected covariates, it cannot be determined whether perceived emotional functioning contributes to more favorable evaluations of life, whether individuals with greater life satisfaction perceive their emotional functioning more positively, or whether both are influenced by unmeasured factors. Reverse or bidirectional relationships therefore cannot be excluded. Accordingly, the findings should be considered associative rather than evidence that emotional intelligence training would improve life satisfaction. Longitudinal and prospective studies are needed to establish temporal relationships, while intervention studies would be required to determine whether changes in emotional functioning produce subsequent changes in life satisfaction.

Second, both perceived emotional intelligence and life satisfaction were assessed simultaneously using self-report questionnaires completed by the same respondents, creating potential for common method variance, response-style bias, and social desirability effects. Although an exploratory Harman's single-factor assessment indicated that the first unrotated component accounted for only 19.9% of the total item-level variance, this procedure cannot rule out common method bias. Future studies should consider temporally separated assessments, multiple information sources, or complementary measurement approaches.

Third, the TMMS-24 is a multidimensional instrument comprising Emotional Attention, Emotional Clarity, and Emotional Repair (Salovey et al., 1995; Fernández-Berrocal et al., 2004, 2025), whereas the prespecified primary analyses in this study used an overall score across the 24 items. The overall score should therefore be interpreted only as the aggregate indicator used in the primary analytical plan and not as evidence that the TMMS-24 is unidimensional or that global scoring is psychometrically preferable to its established three-dimensional structure. Supplementary exploratory analyses were conducted separately for the three dimensions in response to peer review; however, internal consistency was heterogeneous and limited in the present sample (Emotional Attention α = 0.665, Emotional Clarity α = 0.284, and Emotional Repair α = 0.518). Consequently, dimension-specific findings should be interpreted cautiously. Factorial validity and measurement equivalence of the TMMS-24 were not independently established in the present Ecuadorian sample, and measurement invariance across sex or academic program cannot be assumed. These psychometric limitations are particularly relevant to subgroup comparisons and exploratory dimension-specific analyses.

Fourth, the adjusted model included age, sex, and academic program but did not include other potentially relevant psychological, behavioral, academic, and contextual factors, such as perceived stress, anxiety or depressive symptoms, resilience, social support, socioeconomic circumstances, academic performance, or academic workload. Residual confounding and omitted-variable bias therefore cannot be excluded, and the regression coefficients should be interpreted as adjusted associations rather than as unique causal contributions of perceived emotional intelligence to life satisfaction.

Finally, participants were recruited through non-probability sampling at a single Ecuadorian university. Although the participation rate among invited students was high (96.8%), the sampling strategy and single-center design limit representativeness and introduce the possibility of selection bias. The sample therefore cannot be assumed to represent all health sciences students in Ecuador. External validity is consequently limited, and the findings should be generalized cautiously beyond educational settings with comparable student and institutional characteristics. In addition, women represented 80.2% of the sample, and ethnic groups were unequally represented. These characteristics limit the precision of subgroup comparisons and external validity; therefore, the findings should not be generalized beyond similar educational settings without caution. Future multicenter studies should include larger and more balanced samples, examine TMMS-24 dimensions using psychometrically supported scoring approaches, and incorporate a broader range of psychological, social, academic, and socioeconomic factors.

## 5 Conclusion

Perceived emotional intelligence was positively associated with life satisfaction among health sciences students, although the magnitude of the association was moderate and the adjusted model explained a limited proportion of variance. These findings suggest that perceived emotional intelligence represents one of several factors associated with students' life satisfaction rather than a dominant determinant. No statistically significant overall differences in perceived emotional intelligence or life satisfaction were detected across academic programs or ethnic groups. Given the cross-sectional design, the findings should be interpreted as associative rather than causal and do not establish that changes in emotional intelligence would lead to changes in life satisfaction. Supplementary dimension-specific analyses suggested that Emotional Attention remained independently associated with life satisfaction when the three TMMS-24 dimensions were considered simultaneously; however, these analyses were exploratory and constrained by limited subscale internal consistency. They should therefore be considered hypothesis-generating rather than evidence of an established dimension-specific association. Longitudinal, multicenter studies with stronger psychometric support are needed to clarify temporal relationships, dimension-specific associations, and generalizability across educational and cultural contexts.

## Data Availability

The original contributions presented in the study are included in the article/supplementary material, further inquiries can be directed to the corresponding author/s.
